# Lack of SARS-CoV-2-specific cellular response in critically ill COVID-19 patients despite apparent effective vaccination

**DOI:** 10.1186/s13054-022-04038-5

**Published:** 2022-06-08

**Authors:** Frank Bidar, Guillaume Monneret, Franck Berthier, Anne-Claire Lukaszewicz, Fabienne Venet

**Affiliations:** 1 Anaesthesia and Critical Care Medicine Department, Hospices Civils de Lyon, Edouard Herriot Hospital, 69437 Lyon Cedex 3, France; 2 EA 7426 Pathophysiology of Injury-Induced Immunosuppression (PI3), Lyon 1 University/Hospices Civils de Lyon/bioMérieux, Hôpital Edouard Herriot, Lyon, France; 3 Immunology Laboratory, Edouard Herriot Hospital – Hospices Civils de Lyon, 5 place d’Arsonval, 69437 Lyon Cedex 3, France; 4 R&D - Immunoassay, bioMérieux S.A., 376 chemin de l’orme, 69280 Marcy l’Etoile, France; 5 Centre International de Recherche en Infectiologie (CIRI), INSERM U1111, CNRS, UMR5308, Ecole Normale Supérieure de Lyon, Université Claude Bernard-Lyon 1, Lyon, France

Dear Editors,

Since the beginning of the COVID-19 pandemic, messenger RNA vaccination has been highly effective for preventing SARS-CoV-2 infections [1]. However, hospitalizations in intensive care unit (ICU) have been reported among vaccinated patients characterized by advanced age or underlying comorbidities [2, 3]. Little is known about the specific cellular immune response of these patients upon ICU admission although this question is of utmost clinical importance.

In a prospective cohort study, we assessed SARS-CoV-2-specific immune response in 14 ICU patients (7 vaccinated and 7 non-vaccinated) between January and February 2022. Vaccinated patients received at least two doses of BNT162b2 vaccine with a delay ranging from two to six months. Of them, two had pre-existing immunosuppression (one kidney transplant and one mixed connective tissue disease). Demographic and clinical characteristics of the study population are provided in Additional file 1: Table S1. As controls, 8 vaccinated healthy volunteers (HV) were also enrolled.

We evaluated humoral response by measuring anti-Spike IgG titers. Cellular response was assessed by T-cell proliferation assay and whole blood Interferon-Gamma Release Assay (IGRA) after stimulation with SARS-Cov-2 proteins [4].

Upon ICU admission, vaccinated patients presented high anti-Spike IgG titers that were significantly higher than non-vaccinated patients (Fig. 1A). In contrast, T-cell proliferation in response to spike antigen was absent (Fig. 1B). In accordance, in response to SARS-CoV-2 antigens whole blood Interferon-Gamma Release Assay (IGRA) was found to be very low in vaccinated patients whereas they presented with strong response to mitogen (PHA) which illustrated appropriate functionality of T cell (Fig. 1C, D). As controls, HV showed good response in both lymphocyte proliferation and IGRA. Overall, these results indicate that despite apparent successful vaccination (i.e., illustrated by consistent seroconversion), patients admitted to ICU did not develop any cellular response to SARS-CoV-2. Of note, only 2 of 7 vaccinated patients, had previous history of immunosuppression. That said, as previously described in various ICU COVID-19 cohorts, present patients, vaccinated or not, presented with alterations in immune cellular parameters: profound lymphopenia and reduced monocytic HLA-DR expression (Fig. 1E, F).

**Fig. 1:**
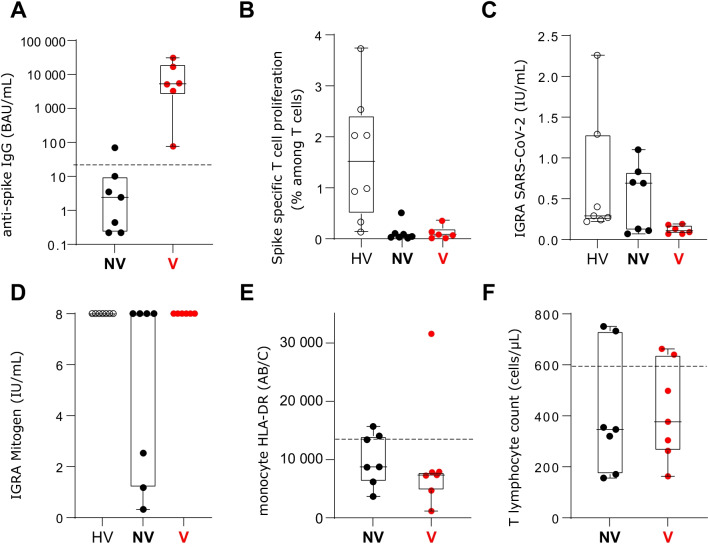
Immune monitoring of vaccinated and non-vaccinated COVID-19 patients at intensive care unit admission. Blood samples were collected within the first 48 h after ICU admission for vaccinated and non-vaccinated COVID-19 patients. Healthy volunteers were enrolled as controls. **A** shows anti-Spike (S1-Receptor Binding Domain) IgG titers measured using the Siemens Atellica IM SARS-CoV-2 IgG (sCOVG) kit and transformed in BAU/mL using the conversion factors provided by the manufacturers. The horizontal dotted line along the x axes indicate the positivity threshold. **B** shows the percentage of CD3 + T-cell proliferating T cells among total T lymphocytes assessed by monitoring EdU AF488 incorporation after stimulation during 7 days with SARS-Cov-2 S1 and S2 peptide pools. **C** shows whole blood Interferon-Gamma Release Assay (IGRA) after specific stimulation with a pool of SARS-CoV-2-specific peptides (bioMérieux Vidas COVIGRA–RUO). **D** shows whole blood Interferon-Gamma Release Assay (IGRA) after mitogen stimulation with phytohemagglutinin. For panels C and D, results are expressed as international units per mL (IU/mL). **E** shows monocyte HLA-DR (mHLA-DR) expression determined using the BD Quantibrite Anti–HLA-DR/Anti-Monocyte standardized method. The horizontal dotted line along the x axes indicates the lowest reference values for mHLA-DR. Results are expressed as numbers of antibodies bound per monocyte (AB/C). **F** shows CD3 + T cell lymphocyte count measured by flow cytometry. The horizontal dotted line along the x axes indicate the lowest reference values for CD3 + T-cell count. Results were expressed as numbers of cells per µL. Results are presented as individual values and Tukey boxplots in healthy volunteers (HV, open dots), non-vaccinated COVID-19 ICU patients (NV, black dots) and in vaccinated COVID-19 patients (V, red dots)

Although obtained in a relatively small cohort of patients, these results suggest that cellular response is also an important determinant of COVID-19 evolution in vaccinated patients. They raise concern about using humoral response as a sole metric of protective immunity following vaccination for SARS-CoV-2 especially in high-risk patients. Specific tools measuring cellular response, usable in a standardized routine practice, could guide the administration of COVID-19 vaccine booster in patients who did not mount any cellular response. This may help preventing evolution towards the most severe forms of the disease. Considering that an uncoordinated T cell and antibody responses have been associated with disease progression [5], the understanding of mechanisms sustaining incomplete vaccination (presence of IgG but lack of T cell response) remains of utmost importance.

## Supplementary Information

**Additional file 1.** Complementary methods and description of clinical data.

## Data Availability

The datasets analyzed during the current study are available from the corresponding author on reasonable request.
