# Supplementary material for: Lack of SARS-CoV-2-specific cellular response in critically ill COVID-19 patients despite apparent effective vaccination
Source: Crit Care. 2022 Jun 8;26:170. doi: 10.1186/s13054-022-04038-5 (PMC9176157; doi:10.1186/s13054-022-04038-5)
Supplement: Supplementary file 1 — Additional file 1. Complementary methods and description of clinical data. [file 13054_2022_4038_MOESM1_ESM.docx]

**Additional file 1**

**Lack of SARS-Cov-2 specific cellular response in critically ill COVID-19 patients despite apparent effective vaccination**

Frank Bidar^1,2^, Guillaume Monneret^2,3^, Franck Berthier^4^, Anne-Claire Lukaszewicz^1,2^, Fabienne Venet^3,5^

1. Anaesthesia and Critical Care Medicine Department, Hospices Civils de Lyon, Edouard Herriot Hospital, 69437 Lyon Cedex 3, France
2. EA 7426 Pathophysiology of injury-induced immunosuppression (PI3), Lyon 1 University/ Hospices Civils de Lyon / bioMérieux, Hôpital Edouard Herriot, Lyon, France
3. Immunology Laboratory, Edouard Herriot Hospital – Hospices Civils de Lyon, 69437 Lyon Cedex 3, France
4. R&D - Immunoassay, bioMérieux S.A., Marcy l'Etoile, France.
5. Centre International de Recherche en Infectiologie (CIRI), INSERM U1111, CNRS, UMR5308, Ecole Normale supérieure de Lyon, Université Claude Bernard-Lyon 1, Lyon, France

**Methods**

*Clinical study design, patient population and ethics approval*

Between January and February 2022, vaccinated and non-vaccinated critically ill COVID-19 patients were included in the RICO (REA-IMMUNO-COVID) cohort. Critically ill patients admitted to two ICUs (Medical and Surgical ICUs of the Edouard Herriot University Hospital) of Lyon university-affiliated hospitals (Hospices Civils de Lyon, Lyon, France) who presented with SARS-CoV-2 pulmonary infection confirmed by RT-PCR were included in the RICO (REA-IMMUNO-COVID) study. This study was approved by ethics committee (Comité de Protection des Personnes Ile de France 1 - N°IRB / IORG #: IORG0009918) under agreement number 2020-A01079-30 and was registered at ClinicalTrials.gov (NCT04392401). The committee waived the need for written informed consent because the study was observational, with a low risk to patients, and no specific procedure, other than routine blood sampling, was required. Oral information and agreement to inclusion in the study were mandatory and were systematically obtained before any blood sample was drawn. This was recorded in patients’ clinical files. If a patient was unable to consent directly, the patient’s legally authorized representative were contacted and agreement was reconfirmed from the patient at the earliest opportunity. Inclusion criteria were: (1) patients aged > 18 years, (2) hospitalization in ICU for SARS-CoV-2 pneumopathy, (3) first hospitalization in the ICU for COVID-19, (4) positive diagnosis of SARS-CoV-2 infection carried out by PCR in at least one respiratory sample, (5) sampling in the first 48h after admission to the ICU feasible and (6) patient or next of kin informed of the terms of the study and has not objected to participate.

Concomitantly, blood samples from 8 vaccinated healthy volunteers were independently obtained from EFS (Etablissement Français du Sang, Lyon, France). Briefly, healthy donors were 53 years old [IQR, 49-58] and were heterogeneously distributed between males and females (2/6).

*Patient characteristics*

For each patient, demographics, comorbidities, time from onset of COVID-19 symptoms to ICU admission, initial presentation of the disease in the ICU were documented. Organ dysfunctions according to Sequential Organ Failure Assessment (SOFA) score (range 0-24, with higher scores indicating more severe organ failures), and Simplified Acute Physiology Score II (SAPS II; range, 0-164, with higher scores indicating greater severity of illness) were documented. Follow-up included ICU length of stay and day-28 (D28) mortality.

**Table S1: Patients’ demographic and clinical characteristics**

Results are expressed as median (IQR) or n (%). BMI: Body mass index, SAPS: Simplified Acute Physiology Score, SOFA: Sequential Organ Failure Assessment.

|  | **Non-vaccinated patients (n=7)** | **Vaccinated patients (n=7)** |
| --- | --- | --- |
| Age (IQR) – year | 58 (57 – 74) | 70 (50 – 72) |
| BMI (IQR) | 27 (25 – 28) | 30 (29 – 34) |
| Gender, Male – no. (%) | 7 (100) | 4 (57) |
| SAPS II (IQR) | 31 (17 – 32) | 33 (25 – 37) |
| SOFA (IQR) | 1 (0 – 3.5) | 2 (1 – 4.5) |
| Mechanical ventilation at admission – no. (%) | 0 (0) | 1 (14) |
| Mechanical ventilation during ICU stay – no. (%) | 1 (14) | 5 (71.4) |
| Day-28 mortality – no. (%) | 1 (14) | 3 (43) |
| Immunosuppression – no. (%) | 0 (0) | 2 (29) |
| Immunosuppressive therapy – no. (%) | 0 (0) | 2 (29) |
| Solid tumor – no. (%) | 1 (14) | 0 (0) |
